# Supplementary material for: Perceptions of COVID-19-related nudges in the Arab world: A cross-country analysis of approval rates and associated factors
Source: PLOS Glob Public Health. 2025 Oct 10;5(10):e0004692. doi: 10.1371/journal.pgph.0004692 (PMC12513628; doi:10.1371/journal.pgph.0004692)
Supplement: S1 Table — (DOCX) [file pgph.0004692.s002.docx]

**S1 Table:** Classification of interventions using the Nuffield Intervention Ladder

| Rank (Least → Most Intrusive) | Intervention Description | Nuffield Category | Justification |
| --- | --- | --- | --- |
| 1 | Public disclosure of infection causes (e.g., hugging) | Provide information | Shares factual information to guide behavior without restricting choice. |
| 2 | Elderly sending letters to family urging compliance | Enable choice | Encourages voluntary compliance through emotional appeal while preserving autonomy. |
| 3 | Supermarket floor markers for social distancing | Change the default | Alters physical environment to make distancing the default, though opt-out remains possible. |
| 4 | Fruits/vegetables displayed first in grocery apps | Change the default | Restructures digital choice architecture to promote healthier selections. |
| 5 | Parks marked with lines to limit group size | Change the default | Structures public space to set implicit norms without legally prohibiting gatherings. |
| 6 | Billboards spoiling TV shows to deter travel | Utilize disincentives | Uses aversive social sanctioning (spoilers) as a deterrent against non-compliance. |
| 7 | Publishing names/nationalities of curfew violators | Utilize disincentives | Creates reputational risk as a non-legal penalty to discourage violations. |
| 8 | GPS tracking app with health & location monitoring | Restrict choice | Introduces surveillance and restricts movement, substantially limiting autonomy. |
