# Supplementary material for: Perceptions of COVID-19-related nudges in the Arab world: A cross-country analysis of approval rates and associated factors
Source: PLOS Glob Public Health. 2025 Oct 10;5(10):e0004692. doi: 10.1371/journal.pgph.0004692 (PMC12513628; doi:10.1371/journal.pgph.0004692)
Supplement: S2 Table — (DOCX) [file pgph.0004692.s003.docx]

**S2 Table.** Approval of COVID-19-related nudges by family COVID-19 infection status

| **Variables** | | **No family members**  **tested positive**  **for COVID-19**  **(n = 181,**  **25.9%)** | **At least one**  **family member**  **tested positive**  **for COVID-19**  **(n = 517,**  **74.1%)** | **p-value** |
| --- | --- | --- | --- | --- |
| **To limit the spread of the coronavirus, an app uses a GPS feature to track users and gather health information about healthy individuals, and those in quarantine.** | **Approve** | 144 (79.56%) | 364 (70.41%) | **0.017** |
|  | **Disapprove** | 37 (20.44%) | 153 (29.59%) |  |
| **To discourage people from all non-essential road travel during the national lockdown, a campaign is launched advertising spoilers of popular television series on billboards.** | **Approve** | 103 (56.91%) | 260 (50.29%) | 0.125 |
|  | **Disapprove** | 78 (43.09%) | 257 (49.71%) |  |
| **To encourage customers to maintain a safe distance of 2 meters between each other, supermarkets are required to install social distancing floor markers at checkout lanes.** | **Approve** | 171 (94.48%) | 495 (95.74%) | 0.482 |
|  | **Disapprove** | 10 (5.52%) | 22 (4.26%) |  |
| **To encourage compliance with the national lockdown rules, the number of people who violate the national curfew and their respective nationalities are publicly published.** | **Approve** | 97 (53.59%) | 251 (48.55%) | 0.243 |
|  | **Disapprove** | 84 (46.41%) | 266 (51.45%) |  |
| **To deter people from gathering in large numbers, popular parks and green areas are divided into squares where no more than 10 people can gather in the same square.** | **Approve** | 147 (81.22%) | 425 (82.21%) | 0.766 |
|  | **Disapprove** | 34 (18.78%) | 92 (17.79%) |  |
| **To increase compliance with social distancing rules, causes of infections are made publicly available (e.g. because of the exchange of hugs and kisses at a family gathering, nine cases of COVID-19 have been detected of which three are being hospitalized).** | **Approve** | 158 (87.29%) | 446 (86.27%) | 0.728 |
|  | **Disapprove** | 23 (12.71%) | 71 (13.73%) |  |
| **To increase compliance with COVID-19 preventive measures, elderly people are asked to send letters to family members pleading with them to respect the rules for their sake.** | **Approve** | 149 (82.32%) | 438 (84.72%) | 0.447 |
|  | **Disapprove** | 32 (17.68%) | 79 (15.28%) |  |
| **To increase healthy eating during the pandemic, grocery stores are required to display fruits and vegetables as the first items on their mobile apps and web shops.** | **Approve** | 159 (87.85%) | 461 (89.17%) | 0.627 |
|  | **Disapprove** | 22 (12.15%) | 56 (10.83%) |  |
