# Supplementary material for: Perceptions of COVID-19-related nudges in the Arab world: A cross-country analysis of approval rates and associated factors
Source: PLOS Glob Public Health. 2025 Oct 10;5(10):e0004692. doi: 10.1371/journal.pgph.0004692 (PMC12513628; doi:10.1371/journal.pgph.0004692)
Supplement: S3 Table — (DOCX) [file pgph.0004692.s004.docx]

**S3 Table**. Approval of COVID-19-related nudges by income

| **Variables** | | **Low**  **N=117** | **Middle**  **N=300** | **High**  **N=171** | **p-value** |
| --- | --- | --- | --- | --- | --- |
| **To limit the spread of the coronavirus, an app uses a GPS feature to track users**  **and gather health information about healthy individuals, and those in quarantine.** | **Approve** | 88 (75.2%) | 228 (76.0%) | 120 (70.2%) | 0.37 |
|  | **Disapprove** | 29 (24.8%) | 72 (24.0%) | 51 (29.8%) |  |
| **To discourage people from all non-essential road travel during the national lockdown, a campaign is launched advertising spoilers of popular television series on billboards.** | **Approve** | 62 (53.0%) | 159 (53.0%) | 76 (44.4%) | 0.17 |
|  | **Disapprove** | 55 (47.0%) | 141 (47.0%) | 95 (55.6%) |  |
| **To encourage customers to maintain a safe distance of 2 meters between each other, supermarkets are required to install social distancing floor markers at checkout lanes.** | **Approve** | 112 (95.7%) | 284 (94.7%) | 168 (98.2%) | 0.17 |
|  | **Disapprove** | 5 (4.3%) | 16 (5.3%) | 3 (1.8%) |  |
| **To encourage compliance with the national lockdown rules, the number of people**  **who violate the national curfew and their respective nationalities are publicly published** | **Approve** | 60 (51.3%) | 155 (51.7%) | 81 (47.4%) | 0.65 |
|  | **Disapprove** | 57 (48.7%) | 145 (48.3%) | 90 (52.6%) |  |
| **To deter people from gathering in large numbers, popular parks and green areas are divided into squares where no more than 10 people can gather in the same square.** | **Approve** | 102 (87.2%) | 236 (78.7%) | 141 (82.5%) | 0.12 |
|  | **Disapprove** | 15 (12.8%) | 64 (21.3%) | 30 (17.5%) |  |
| **To increase compliance with social distancing rules, causes of infections are made publicly available (e.g. because of the exchange of hugs and kisses at a family gathering,  nine cases of COVID-19 have been detected of which three are being hospitalized).** | **Approve** | 104 (88.9%) | 260 (86.7%) | 146 (85.4%) | 0.69 |
|  | **Disapprove** | 13 (11.1%) | 40 (13.3%) | 25 (14.6%) |  |
| **To increase compliance with COVID-19 preventive measures, elderly people are asked to send letters to family members pleading with them to respect the rules for their sake.** | **Approve** | 102 (87.2%) | 250 (83.3%) | 140 (81.9%) | 0.48 |
|  | **Disapprove** | 15 (12.8%) | 50 (16.7%) | 31 (18.1%) |  |
| **To increase healthy eating during the pandemic, grocery stores are required to display fruits and vegetables as the first items on their mobile apps and web shops.** | **Approve** | 105 (89.7%) | 273 (91.0%) | 150 (87.7%) | 0.53 |
|  | **Disapprove** | 12 (10.3%) | 27 (9.0%) | 21 (12.3%) |  |
