# Supplementary material for: Perceptions of COVID-19-related nudges in the Arab world: A cross-country analysis of approval rates and associated factors
Source: PLOS Glob Public Health. 2025 Oct 10;5(10):e0004692. doi: 10.1371/journal.pgph.0004692 (PMC12513628; doi:10.1371/journal.pgph.0004692)
Supplement: Attachment — Submitted filename: Response to Reviewers.docx [file pgph.0004692.s006.docx]

**Response to Reviewers**

**Additional Editor Comments (if provided):**

Thank you very much for your submission. Please refer to reviewer's comments and address all of them. There are no contradictory comments in the reviewer's suggestions that should cause confusion in the revision of the manuscript. Pay particular attention to PLOS Global Public Health policy regarding data availability.

- Reply: Thank you for the clarification. Please note we have uploaded the data set used in our study to the OSF repository.
- <https://osf.io/eav4j/?view_only=4f8c85081ef347e7853ea9f23e4480d0>
- At the moment, the project is set to private for confidentiality reasons but will be made public once the paper is published.

In addition to reviewers' comments, authors should refrain from conclusions or interpretation not sustained by the study. For instance, since the effectivity of the actual nudges was not evaluated, it can hardly be advisable for policy-makers to implement a nudge strategy based only on the results from this study.

- Reply: Thank you for this important suggestion. We agree that our conclusions should reflect what our data can support. We have revised the relevant portions of the discussion section to avoid suggesting policy action based solely on our findings, and we now more clearly emphasize the hypothetical nature of the nudges and the need for further real-world research (such as experimental or observational studies) to assess their effectiveness before policy implementation is considered.

**Comments to the Author**

**Reviewer #1:** Thank you for the opportunity to review this manuscript. The study on COVID-19-related nudges is interesting, given the severity of the crisis and the cultural context of the Arab world. Here are my comments, which I hope will be useful for your revision.

First, I believe that the inclusion of data from the four Arab countries (Egypt, Lebanon, Saudi Arabia, and the UAE) strengthens the generalizability of the findings within the region. That said, because studies on nudges have generally come from the West, and you employ methodologies from such studies, you should enhance the discussion on its comparisons to the existing literature, which you only do so in brief (e.g., Sunstein et al.). In particular, expand the discussion on why certain nudges (e.g., GPS tracking) are more accepted in the Arab world than in Western contexts, possibly due to differing attitudes toward privacy and public health priorities. This discussion will highlight the importance of understanding variations in public perceptions based on cultural and national differences when developing nudge interventions.

- Reply: Thank you for your feedback. We have now modified our Discussion section to address this point. We have added the following paragraph: “Our findings on the relatively high approval of intrusive nudges, such as GPS tracking (72.8%), contrast with previous studies conducted in Western contexts, where similar interventions have typically received lower acceptance due to heightened privacy concerns [16, 17]. This divergence likely reflects deeper cultural differences in societal attitudes toward individual autonomy, privacy, and governmental authority. Arab societies, often characterized by collectivist values and stronger emphasis on community responsibility, might prioritize public health and collective safety over individual autonomy during crises [8]. Additionally, variations in trust towards government and public health authorities between Arab and Western contexts may further explain the greater acceptance of measures like GPS tracking observed in our study [18]. These results align with cultural theories such as Hofstede’s dimensions [8], highlighting the critical importance of cultural context when designing and implementing behavioral interventions.”

Your theory development could be stronger. You used Hofstede’s cultural dimensions but do not connect those dimensions to the nudges. Please discuss how specific cultural dimensions (e.g., power distance, uncertainty avoidance, etc.) explains the differences in the acceptance of intrusive versus instructive nudges. Also, please note that Hofstede's dimensions have been criticized for its methodology and generalizability (Venaik, S. and Brewer, P. (2013), "Critical issues in the Hofstede and GLOBE national culture models", International Marketing Review, Vol. 30 No. 5, pp. 469-482. https://doi.org/10.1108/IMR-03-2013-0058). You may want to address this in your methods and say why the framework is appropriate for this study.

- Reply: Thank you for the comment. We have modified our paper to include the reasoning behind the choice of this framework for this study. This is modified in the Introduction section as: “Hofstede's cultural dimensions theory presents a valuable framework for this study [8]. It provides a structured lens to examine how cultural dimensions such as power distance, individualism, uncertainty avoidance, and indulgence may shape nudge acceptability in the Arab world, where collectivism and hierarchical social structures are prominent [9, 10]. Despite receiving criticisms [11], Hofstede’s framework fits well with the aim of this paper, which is to demonstrate how similar nudges can have different reactions depending on the differences in the cultural dimensions of societies” and again expanded on in the Nudge Types section, starting from “Finally, the types of nudges selected also align well with Hofstede’s cultural index…”

The sample consists primarily of university students and academic staff, which may not fully represent broader demographic groups in the Arab world. At minimum, you should acknowledge this limitation and discuss how future studies could include more diverse participants (e.g., older adults, rural populations, lower-income groups). You can also try to address this limitation with sub-group analyses by the demographic variables in your data to determine if there are systematic biases.

- Reply: Thank you for your comment. We appreciate your feedback and have now modified our limitation section. It now reads as follows: “While our study provides valuable insights, it has several limitations. The sample was predominantly composed of university students and staff, which may not fully represent the broader Arab population, particularly older adults, rural communities, and lower-income groups. To partially address this limitation, we conducted subgroup analyses based on key demographic and contextual variables, including age, gender, degree of concern about COVID-19, and family COVID-19 status. Future research should aim to include more demographically diverse participants across various socioeconomic and geographic strata to enhance generalizability.”

I do not understand how you categorized the nudges into intrusive, instructive, informative, entertainment, and moral. Please discuss how you derived these categories with a stronger theoretical or empirical basis for the classifications, possibly referencing the literature.

- Reply: Thank you for your comment. We have modified the Nudge Types section to incorporate the justification of the choice of each type of nudge that was based on Hofstede’s cultural index. The modified section starts from: “In this study, we selected eight nudges commonly used in the literature...”

Other variables, such as trust in government, and exposure to misinformation, or religious beliefs, may influence nudge approval but are not examined. These are potential confounders and should be addressed in how future research should explore their role in shaping public attitudes toward nudges.

- Reply: Thank you for your comment. We acknowledge that variables such as trust in government, exposure to misinformation, and religious beliefs may play an important role in shaping public attitudes toward nudges and represent a limitation of our study. Due to the sensitivity and complexity of measuring these variables, we did not include them in the current analysis. However, we agree that future research should explore their potential influence as important contextual and cultural factors. We have now modified our discussion section to acknowledge this as a limitation of our study. It now reads as follows: “Moreover, we did not include variables such as trust in government, exposure to misinformation, or religious beliefs, which may significantly influence attitudes toward nudges. These factors are complex and sensitive to measure but represent important cultural and contextual dimensions that should be explored in future research.”

My biggest concern with this study is that it evaluates hypothetical nudges rather than real-world interventions. Hence, there is a limit to what we can learn from this paper. You might want to explore further how perceptions of nudges might differ if implemented in real settings and suggest avenues for experimental or observational studies. This weakness makes the policy discussion less meaningful. While you mention the importance of considering cultural sensitivities, you can't provide specific recommendations for designing and implementing nudges in Arab countries.

- Reply: Thank you for your comment. We fully agree that we evaluated hypothetical nudges and this can present limitations, and we acknowledge that attitudes may not always translate into real-world behaviors. However, this study was designed as an initial exploratory step to assess public acceptability of different types of nudges within the cultural context of Arab countries. We believe these findings can provide foundational insights for future research, including experimental or observational studies that evaluate the real-world implementation and effectiveness of these interventions. To address your concern, we have revised the discussion section to more clearly acknowledge this limitation and to highlight potential avenues for future research, including real-world implementation studies and the exploration of behavioral outcomes.

**Reviewer #2:** This study examines public approval of COVID-19-related nudges in Egypt, Lebanon, Saudi Arabia, and the UAE. Using survey data from 698 participants, it finds high overall support (76%), especially for informative and instructive nudges. Intrusive nudges were less favored. Approval varied by country, demographics, and level of COVID-19 concern. The findings suggest nudging can be effective in the Arab world if culturally tailored.

This is an interesting paper that presents novel findings from a region where public opinion data—particularly on behavioral interventions like nudges—is relatively scarce. The cross-country design and focus on COVID-19-related nudges in the Arab world are valuable contributions. The results are clear and relevant for both policy and behavioral science. That said, the paper would benefit from addressing several issues outlined below.

**Comments:**

1. In the introduction, the authors state that “the effectiveness of nudging has been well-documented in Western contexts.” This phrasing may imply that nudges are always effective, which is not the case. I recommend rephrasing to acknowledge the mixed evidence and contextual variability in nudge effectiveness.

- Reply: Thank you for the comment. We have modified this section and now it reads as follows: “The testing of nudging has been well-documented in various Western contexts; however, empirical evidence indicates that its impact varies across different behavioral domains and settings. A comprehensive meta-analysis by Mertens et al. (2022) found that while nudging interventions generally produced positive effects, the magnitude of these effects varied significantly across different behavioral domains, ranging from small to moderate effect sizes (Cohen’s d = 0.24 to 0.65) [6].”

2. The Materials and Methods section does not explain how participants were recruited or how the survey was distributed. Was it via flyers, social media, email lists, or another method? These details are important for assessing sample representativeness and potential biases.

- Reply: Thank you for your comment. We have modified the Materials and Methods section to include this information. We have added this additional sentence: “The survey was distributed electronically via email to all university affiliates, including students, faculty, and administrative staff. Participation was voluntary, and no incentives were provided.”

3. The authors state that informed consent was obtained while maintaining anonymity. More detail is needed: how was consent collected in practice (e.g., online checkbox, written form)? How was anonymity preserved during this process?

- Reply: Thank you for your comment. We have now modified the Materials and Methods section to reflect this. We added this additional paragraph: “Informed consent was obtained online through an electronic consent form presented at the beginning of the survey. Participants were informed that their participation was voluntary and that their responses would remain anonymous and confidential. Only those who actively indicated consent (by selecting a checkbox) were able to proceed with the survey. Anonymity was maintained by collecting only non-sensitive, non-identifiable demographic data, with no names, email addresses, or other personal identifiers recorded. To ensure confidentiality, access to the survey data was restricted to the principal investigators and the statistician involved in the analysis.”

4. The approval/disapproval measure is binary. The authors should briefly explain why this approach was chosen over a Likert-type or continuous scale, which could capture more nuanced attitudes.

- Reply: Thank you for your feedback. We chose a dichotomous scale to simplify interpretation of results and facilitate comparisons across subgroups. This decision was also taken due to the relatively small sample sizes in certain subgroups of these survey questions, which may have limited the reliability of Likert-type or continuous measures. We acknowledge that a more nuanced scale could provide additional insight, and future studies with larger and more diverse samples may benefit from employing such measures.

5. Some categorizations are unclear. For example, the prominent display of fruits and vegetables is labeled as “informative,” though it may be more accurately classified as environmental or design-based. The “spoilers” nudge is categorized as “entertainment,” but that label may not effectively convey its behavioral mechanism. Please clarify the reasoning behind these classifications.

- Reply: Thank you for the comment. We have modified the Nudges Types section to incorporate the justification of the choice of each type of nudge that was based on Hofstede’s cultural index. The modified section is: “In this study, we selected eight nudges commonly used in the literature...”

6. There should be more discussion about how the eight nudges were selected. For instance, the fruits and vegetables display is not COVID-related—why was it included? Were any of the nudges based on actual policies implemented in the studied countries? If so, noting this would strengthen the paper.

- Reply: Thank you for your feedback. First, we would like to clarify that the nudges in our study are a combination of interventions we observed and illustrative examples. Several of these were indeed inspired by actual measures implemented by governments and institutions during the COVID-19 pandemic. Examples include GPS tracking, social distancing floor markers, and the public disclosure of infection causes, all of which were observed in various countries across the Middle East. Second, our selection of interventions was intentional to cover a range of intrusiveness, from subtle nudges like product placement to more overt strategies such as public shaming for curfew violations. Our aim was to examine public acceptance of COVID-19 policies, as well as perceptions of behavioral influence during a crisis. To ensure a balanced and contextually relevant set, we consulted regional and behavioral science experts who assessed the options based on their perceived intrusiveness and relevance to the pandemic in the context of the region. Concerning the healthy eating intervention, we understand that its connection to COVID-19 might seem less direct. However, its inclusion was based on two main reasons. Firstly, promoting healthy eating was widely encouraged by public health authorities during the pandemic as a way to improve immunity and resilience against infection. Secondly, our team has practical experience in designing and implementing healthy eating interventions in collaboration with Health Ministries, the World Health Organization, and universities in Lebanon and Qatar, both during and before the pandemic. This real-world experience supported the inclusion of this intervention in our study for its plausibility and relevance. We have now revised our Materials and Methods section/Nudges types section to address these points.

7. It would be helpful to report the distribution of the total number of nudges approved by respondents, both overall and by country. This aggregate measure could also serve as a useful dependent variable in additional analyses.

- Reply: Thank you for your comment. We have realized that we originally had a table showing the approval rates for the individual nudges but was left out by mistake. We have now included it again as Table 2. Although our original results section included overall approval rates per country, we have since revised the section to avoid reporting or comparing the total number of nudges approved per country. This decision was made to prevent potential political sensitivities that may arise from direct country-by-country comparisons. Additionally, we acknowledge that our sample sizes are not proportionally representative of the population sizes across the four countries studied, which further limits the appropriateness of such comparisons. We have now emphasized general trends without engaging in direct cross-country comparisons.
