# Supplementary material for: Perceptions of COVID-19-related nudges in the Arab world: A cross-country analysis of approval rates and associated factors
Source: PLOS Glob Public Health. 2025 Oct 10;5(10):e0004692. doi: 10.1371/journal.pgph.0004692 (PMC12513628; doi:10.1371/journal.pgph.0004692)
Supplement: Attachment — Submitted filename: Response to Reviewersv2.docx [file pgph.0004692.s007.docx]

**Response to Reviewers**

**1. Participant recruitment and sample representativeness**
Partly addressed. While the authors now mention that recruitment was conducted via institutional mailing lists, they do not report how many emails were sent, what the response rate was, or whether the sample is representative of the broader target population. This information is important for assessing potential selection bias and the generalizability of the results. I recommend including these additional details.

**Authors’ Response:**

Thank you for your feedback. Recruitment was conducted through institutional mailing lists, with each participating institution distributing one initial invitation email followed by two reminder emails over the course of the recruitment period. We have now added the number of responses received from each university and included estimated response rates based on publicly available population sizes. This information is presented in the Results section. We also acknowledged the potential for selection bias due to low response rates and limited representativeness, and this limitation is now explicitly discussed in the revised Discussion section.

**2. Unclear categorization of nudges**
Not addressed. Several categorizations remain unclear or inadequately justified. For example, the prominent display of fruits and vegetables continues to be labeled as “informative,” whereas this nudge is better understood as a salience-based or choice architecture intervention aimed at influencing defaults or attention. Similarly, the classification of the TV spoiler intervention as “entertainment” fails to capture its behavioral mechanism—namely, deterrence through aversive social sanctioning.
I strongly encourage the authors to revise the typology of nudges using more analytically grounded behavioral categories, such as:
-Choice architecture / salience
-Information disclosure / feedback
-Emotional or moral appeals
-Social norm enforcement / sanctions
-Coercive or surveillance-based interventions
Reclassifying nudges in this way (or in a similar way) would improve conceptual clarity and strengthen alignment with established behavioral economics frameworks.

**Authors’ Response:**

Thank you for this insightful and constructive recommendation. We agree that our original categorization of nudges, based primarily on content or context (e.g., “informative,” “entertainment”), did not fully capture the underlying behavioral mechanisms. In line with your suggestion, we have re-classified the eight nudges into five analytically grounded categories drawn from the behavioral-economics literature:

1. Choice architecture

2. Information disclosure

3. Moral appeals

4. Social norm enforcement (including deterrence through aversive social sanctioning)

5. Surveillance-based interventions

For example, the prominent display of fruits and vegetables is now placed under “choice architecture”, and the use of spoilers on billboards is treated as deterrence within “social norm enforcement”. These revised classifications have been applied consistently throughout the manuscript.

Because the full rationale for this typology is conceptually dense, we have moved the detailed explanation from the Methods to the Discussion, where it is better suited and allows readers to see how the categories align with cultural theory and previous research.

**3.** **Selection and justification of nudges**
Not addressed. The rationale for including the eight specific nudges remains unclear. While the authors reference Hofstede’s cultural dimensions, they do not explain how these dimensions guided the selection of nudges.
Regarding the healthy eating nudge (display of fruits and vegetables), which is not specific to COVID-19, the authors now state that healthy eating was promoted during the pandemic to improve immunity in the studied countries, but they do not provide citations or supporting evidence. A reference or link to relevant policy guidance or public communications would be helpful to support this claim.

**Authors’ Response:**

Thank you for this valuable comment. We have revised the discussion section to clearly articulate the rationale for selecting each of the eight nudges, including how Hofstede’s cultural dimensions informed the selection of behavioral mechanisms. For example, nudges involving hierarchical authority and compliance (e.g., surveillance and public shaming) were included based on the high power distance scores observed in Arab societies. Nudges that promote structure and predictability (e.g., floor markers and infection disclosure) were aligned with high uncertainty avoidance, while moral appeals (e.g., elderly letters) were selected to reflect low individualism and strong familial values.

Regarding the inclusion of the healthy eating nudge, we agree that additional justification was needed. While this intervention is not unique to the pandemic, several ministries of health across the region, as well as the WHO Eastern Mediterranean Regional Office (EMRO), issued public guidance during COVID-19 encouraging healthy diets to support immune function and overall resilience. We have now included citations to relevant policy guidance and WHO EMRO recommendations, and these have been incorporated into the updated discussion.
