# Supplementary material for: Perceptions of COVID-19-related nudges in the Arab world: A cross-country analysis of approval rates and associated factors
Source: PLOS Glob Public Health. 2025 Oct 10;5(10):e0004692. doi: 10.1371/journal.pgph.0004692 (PMC12513628; doi:10.1371/journal.pgph.0004692)
Supplement: Attachment — Submitted filename: Response to editor.docx [file pgph.0004692.s008.docx]

**Response to Editor and Reviewer Comments**
Manuscript ID: PGPH-D-25-00340R2
Title: *Perceptions of COVID-19-related nudges in the Arab world: A cross-country analysis of approval rates and associated factors*

Dear Editor,

We thank you for the thorough and constructive feedback provided on our manuscript. Below we provide a point-by-point response to each comment. All changes are highlighted in the tracked-changes version of the revised manuscript.

1. In the methods and/or conclusions of the abstract, please indicate that the findings apply to university settings and not the general public.

We revised the final two sentences of the Abstract to state that the findings apply to the university setting and may not be generalizable to the wider public. The text now reads: ”*These findings demonstrate generally positive attitudes towards COVID-19-related nudges among university affiliates in four Arab countries, highlighting important variations based on behavioral mechanism and sociodemographic factors. While the results offer valuable insights for culturally tailored behavioral interventions in the Arab world, they reflect a university setting and may not be generalizable to the broader public.”*

2. Specify what stringent measures where used to maintain participant anonymity and data confidentiality (pages 123-124).

The information on anonymity and confidentiality was already provided in the preceding Data collection paragraph:“*Anonymity was maintained by collecting only non-sensitive, non-identifiable demographic data, with no names, email addresses, or other personal identifiers recorded. To ensure confidentiality, access to the survey data was restricted to the principal investigators and the statistician involved in the analysis.”*

For clarity and to avoid redundancy, we have now deleted the duplicate sentence “Stringent measures were implemented to maintain participant anonymity and data confidentiality” from the Ethical considerations section.

3. Line 127. Specify which Supplemental Information is being references (currently it reads "SX").

Replaced “(SX Checklist)” with “(S1 Inclusivity in Global Research Checklist).”

4. Specify in methods/results whether data on university affiliation (i.e., student, faculty, staff) was collected; if so, whether it was analyzed; and, if so, whether it had any impact on outcomes.

We have now included Table 6 to show this data.

5. Denote sample size with "n" and universe size with "N."

We have revised all tables to ensure that sample sizes are denoted with lowercase n and the total sample size is denoted with uppercase N.

6. Lines 162-165 state that country (Lebanon, UAE< Saudi Arabia or Egypt) are denoted in Table 1, but Table 1 does not show that information. Please update Table 1.

We have retained Table 1 without adding a separate “Country” row to avoid redundancy, as the country of each participating institution is already explicit in the text. However, we have revised the Results – Sociodemographic characteristics paragraph to clearly link each institution to its respective country and to summarise participant distribution by country. The text now reads: “ *Our study included 698 participants from four Arab countries. Responses by institution were: 106 from Alfaisal University (Saudi Arabia; 15.2%), 90 from the American University in Cairo (Egypt; 12.9%), 386 from Beirut Arab University (Lebanon; 55.3%), and 116 from the University of Sharjah (United Arab Emirates; 16.6%).”*

7. Lines 165-166, assure consistence between what is reported in the text and what is reported in the table. Suggest to report only the most appropriate measures of central tendency and dispersion (i.e., mean and SD OR median and IQR) as opposed to both. Same for number of children in Table 1.

We have now ensured full consistency between the text and Table 1. For Age and Number of children, we now report only the most appropriate measure of central tendency and dispersion—median and interquartile range (IQR)—given the skewed distribution of these variables. Mean and standard deviation (SD) values for these variables have been removed in the revised Table 1.

8. In Table 1, report BMI only once (i.e., either as categories OR as "BMI = Healthy").

We have revised Table 1 to report BMI only once by retaining the BMI categories (underweight, healthy, overweight, obese) and removing the separate “BMI = Healthy” row to avoid redundancy.

9. Table 1, add "self-reported" where applicable.

We have added the label “(self-reported)” in Table 1 for variables that were based on participant self-report, including BMI and chronic illness

10. Explain definition, measurement and meaning of the following variable: "how would you describe your current health"?

We have revised the Methods – Survey instrument section to define and explain the measurement of the “self-health” variable. The text now reads: “*The second section focused on health-related factors, including self-reported health status, measured by the question “How would you describe your current health?” on a 7-point Likert scale (1 = very poor, 7 = excellent)*. *”* This was also added to Table 1 caption.

11. In Table 1, report "self-health" and "COVID concern" only once.

We have removed duplicate reporting of “self-health” and “COVID concern” in Table 1. Both variables are now listed once, with their definitions included in the table caption for clarity.

12. Line 180, suggest to delete "the" before "choice."

We have removed the word “the” before “choice” in the Results section as suggested.

13. In the methods section, "prominent placement of fruits and vegetables" is listed under the "choice architecture" category (Lines 140-141) ) whereas it is listed under the "information disclosure" category (Lines 182-184). Ensure that it is listed under the right category in both occasions.

We have ensured that “prominent placement of fruits and vegetables” is consistently categorised under choice architecture in both the Methods – Nudge types section and the Results – Nudge approval rates section , correcting earlier inconsistencies.

14. Line 184, suggest to delete "the" before "moral." Also, suggest to write "Moral nudges" as opposed to "Moral nudge."

We have removed the article “the” before “moral” and changed the wording to “Moral nudges” in the Results section to align with the reviewer’s suggestion.

15. Need to justify how "prominent placement of fruits and vegetables" is a "COVID-19-related nudge."

This point was addressed in the previous round of review: (“*Although the prominent display of fruits and vegetables in grocery apps is not COVID-specific, it was included as a choice architecture nudge because several ministries of health in the region, along with the WHO Eastern Mediterranean Regional Office (EMRO), issued guidance during the pandemic encouraging healthy eating to support immune function and reduce vulnerability to infection.)*

16. Explain what is meant by "use of spoilers on billboards."

We have clarified the meaning of “use of spoilers on billboards” in the Methods – Survey instrument section. The text now reads: (“*social norm enforcement (public shaming of violators and use of spoilers on billboards, involving the public display of plot details from popular television series during lockdowns to discourage non-essential travel.*)

17. Explain under what category of nudges does "use of spoilers on billboards" fall into?

We have clarified in the Methods – Survey instrument section that “use of spoilers on billboards” falls under the social norm enforcement category of nudges.

18. Table 2, ensure formatting is correct.

We have reviewed and corrected the formatting of Table 2.

19. Explain in text what "park area divisions" mean.

We have revised the Methods – Survey instrument section to include a definition of “park area divisions.” The text now reads: “… (2) *park area divisions limiting the number of people per section (e.g., a maximum of 10 individuals per square), achieved by demarcating green spaces into marked areas).*”

20. Table 2, identify which category each nudge belong to.

We have revised Table 2 to explicitly identify the category for each nudge and added a new column showing its intrusiveness rank based on the Nuffield Council on Bioethics Intervention Ladder. This allows readers to compare approval not only by category but also by the relative level of intrusiveness.

21. Table 2, if not verbatim and explicitly described in the text of the nudge description, identify in parentheses, what type of nudge it is (i.e., public shaming, etc.).

As indicated in our response to Comment 20, we have added a dedicated ‘Category’ column in Table 2. In addition, we included an ‘Intrusiveness rank’ column, which orders nudges from 1 (least intrusive) to 8 (most intrusive) according to the Nuffield Ladder.

22. Line 198, p<0.05 also found for the "nationalities being published" nudge.

We have revised the Results – Factors associated with nudge approval section to indicate that both the spoilers on billboards and the nationalities being published nudges showed statistically significant differences by age.

23. Explain how "COVID concern" was measured. What instrument was used? What do the scores mean?

We have revised the Methods – Survey instrument section to clarify how “COVID concern” was measured. The text now reads: “……*COVID-19 concern level, measured on a 7-point Likert scale (1 = not at all concerned, 7 = extremely concerned”.*

24. Line 204, there were two additional nudges showing p<0.05 associations with "COVID concern."

We have revised the Results – Factors associated with nudge approval section to explicitly report all statistically significant associations with COVID-19 concern shown in Table 4. We have added this sentence: “*Approval was also higher among high-concern participants for publication of curfew violators’ nationalities (57.4% vs. 41.6%, p < 0.001) and elderly letters (87.6% vs. 80.2%, p = 0.008).”*

25. Lines 205-209, add percentages of approval. Also, add table showing this data.

We have revised the Results – Factors associated with nudge approval section to include the percentages of approval and Table 5 (gendre) & table 6 (occupation), Table S3 (family member tested positive for COVID-19) and Table S4 (Income).

26. Report associations with other variables, for instance, faculty vs. student, income level, medicine/health vs. other (do you have information about whether participants are affiliated with an economics and/or behavioral science department - if so, analyze and report associations), etc..

Our dataset included information on occupation (student vs. faculty/administrative staff), income level (low, middle, high), and academic major (medicine, health, or non-health). However, we did not collect data on specific departmental affiliations (e.g., economics or behavioral science). As mentioned earlier we have added 2 tables in main manuscript and 2 in the supplement to show the additional data.

27. Lines 222-225, specify that this data comes from a university setting, not the general population.

We have revised the Discussion to specify that the findings come from a university setting and are not representative of the general population. The text now reads:

“*This study provides novel insights into the perception and approval of COVID-19-related nudges across four Arab countries in a university setting. Our findings reveal a generally high level of approval for nudges, ranging from 49.9% to 95.4%. However, we observed significant variations in approval rates depending on the type of nudge and sociodemographic factors, and these results should not be assumed to represent the general population.”*

28. Was being faculty vs. student have an impact on COVID?

Yes, occupation (faculty/administrative staff vs. students) was associated with differences in approval of certain nudges. Specifically, faculty/staff were significantly more likely than students to approve of billboard spoilers (61.0% vs. 47.3%, p < 0.001) and public shaming through publishing nationalities of curfew violators (55.6% vs. 46.8%, p = 0.03). For the other six nudges, no statistically significant differences were observed between the two groups. These results are presented in Table 6.

29. Line 227, do you mean "acceptability" instead of "acceptance". Also, suggest to add "nudge-based" before intervention.

We have revised line 227 to replace “acceptance” with “acceptability” and to add “nudge-based” before “interventions.”

30. Line 238, consider deleting "established."

We deleted established.

31. Line 243, clarify whether those (i.e., GPS tracking, social distancing floor markers, and public disclosure of infection causes) are examples of "observed interventions," "illustrative examples," or "actual implementation." Also, how do you distinguish between "observed interventions" and "actual implementation."

The interventions we describe at line 243, GPS tracking, social distancing floor markers, and public disclosure of infection causes, are examples of actual implementations observed in practice across several Arab countries during COVID-19. In our study, we use the term *observed interventions* to mean documented, real-world measures that governments adopted (as opposed to purely hypothetical or illustrative examples). To avoid ambiguity, we have revised the text to explicitly state that these were “implemented” interventions and ensure consistency in how we distinguish between “observed” (i.e., implemented and recorded in the field) and “illustrative” (i.e., used as conceptual examples only).

32. 249, this aim is different than the one stated at the beginning of the paper (i.e., nudges vs. policies). Be consistent.

We have revised the aim statement in the Discussion to ensure consistency with the Introduction, focusing specifically on nudges rather than policies. The text now reads:

…. *to assess public perceptions and acceptability of nudge-based COVID-19-related interventions..”*

33. Lines 240-252, this process of selecting nudges should be moved to the methods section. Also, explain on more detail how experts where consulted and how they provided feedback. Was there a particular methodology used?

We have moved the description of the nudge selection process into a dedicated paragraph in the Methods section called: “Nudge selection”.

Our consultation with experts was informal. We shared a preliminary list of interventions with regional and behavioral science experts, who provided feedback on their perceived intrusiveness and relevance to the pandemic through brief discussions and written comments. Their input helped refine the final selection of interventions.

To clarify this, we have now added the following text : “*To ensure contextual appropriateness, we sought feedback from regional and behavioral science experts, who reviewed a preliminary list and provided informal input on perceived intrusiveness and pandemic relevance. Their feedback informed the refinement of the final set of interventions.”*

34. Explain, in the methods, how did the Hofstede's scale informed nudge selection. Despite being "high power distance" some of the nudges selected range at different levels of power balance/hierarchy/intrusiveness. Did Hofstede's scale play a role in nudge selection? in the analysis? or only in the discussion?

Hofstede’s cultural dimensions framework was not used as a selection criterion for the nudges in the methods. Rather, it was drawn upon later in the discussion to interpret how certain nudges may resonate differently in high power-distance contexts. The selection of nudges was based primarily on expert feedback regarding perceived intrusiveness and relevance to the COVID-19 pandemic. To systematically classify intrusiveness, we applied the Nuffield Council on Bioethics Intervention Ladder, assigning each intervention a rank from 1 (least intrusive) to 8 (most intrusive). This ranking is now displayed in Table 2 alongside approval rates, while full justifications are retained in Supplementary Table S1. We also revised the Results and Discussion sections to explicitly report approval patterns by intrusiveness rank as well as by category.

35. Lines 266-270, please move to methods section where nudges and their selection are being explained.

We have moved lines 266–270 to the Methods section.

36. Line 304 needs rephrasing, check for grammar and syntax.

We have rephrased line 304 in the Discussion to improve grammar and syntax. The text now reads:“*The higher approval rates among female participants for nudges such as GPS tracking and floor markers corroborate the findings of Ölander and Thøgersen, who reported gender differences in receptiveness to pro-environmental nudges*.”

37. Lines 319-320, move up, next to 312.

Done.

38. Line 329, defined the target population, put that definition in the introduction and/or methods.

We have defined the target population and added this clarification to both the Introduction and the Methods – Study design and participants sections. The text now reads:

Introduction: “… *The target population comprised university affiliates, including students, faculty, and administrative staff, from the four participating institutions*.”

Methods: “*The target population was university affiliates (students, faculty, and administrative staff) at the four participating universities. A total of 698 participants were recruited through partner universities in each country*…”

39. Line 333-334, this sub-category analysis is still limited by the sample being taken from a university setting, it does not address the limitation.

We have revised the Discussion – Limitations section to clarify that the subgroup analyses remain limited because the data were collected exclusively from a university setting. We have added this sentence :“…. *However, these subgroup analyses remain limited by the fact that the data were drawn exclusively from a university setting and may not reflect the perspectives of the general population*.”

40. Line 349-351, indicate that the population studied was in a university setting.
We have revised the Conclusion to specify that the findings are based on a university setting. We have added the sentence:“ *These findings reflect the perspectives of university affiliates and may not be generalizable to the broader population*.”

GENERAL: not clear if the "public disclosure" nudges disclose aggregated data OR individual, identifiable data with, say, cause of infection or nationality. Please clarify this in the methods section.

We thank the reviewer for this observation. To clarify, the “public disclosure” interventions in our study refer to the reporting of aggregated and anonymized data, not the release of individually identifiable information. For example, Ministries of Health in Saudi Arabia, UAE, Qatar, and other countries reported during daily briefings that new infections were linked to particular settings or behaviors (e.g., family gatherings, weddings, or workplaces). We have revised the Methods section to explicitly note that these disclosures involved anonymized, setting-level information rather than personal identifiers.

We believe these changes address all comments comprehensively and improve the clarity, rigour, and transparency of the manuscript.

We appreciate the opportunity to revise our work and look forward to your consideration of this updated version.
